# High mammographic density in women is associated with protumor inflammation

**DOI:** 10.1186/s13058-018-1010-2

**Published:** 2018-08-09

**Authors:** Cecilia W. Huo, Prue Hill, Grace Chew, Paul J. Neeson, Heloise Halse, Elizabeth D. Williams, Michael A. Henderson, Erik W. Thompson, Kara L. Britt

**Affiliations:** 1 Department of Surgery, St. Vincent’s Hospital, University of Melbourne, Melbourne, Australia; 2 Department of Pathology, St Vincent’s Hospital, Melbourne, Australia; 3 Pathology Department, University of Melbourne, Melbourne, Australia; 4 Peter MacCallum Cancer Centre, Melbourne, Australia; 5 Institute of Health and Biomedical Innovation and School of Biomedical Sciences, Queensland University of Technology, Brisbane, Australia; 6 Translational Research Institute, Brisbane, Australia; 7 The Sir Peter MacCallum Department of Oncology, University of Melbourne, Melbourne, Australia

**Keywords:** Mammographic density, Immune infiltration, Macrophages, Dendritic cells, PD-L1, B cells, T cells, Biomarker, Immunotherapy

## Abstract

**Background:**

Epidemiological studies have consistently shown that increased mammographic density (MD) is a strong risk factor for breast cancer. We previously observed an elevated number of vimentin^+^/CD45^+^ leukocytes in high MD (HMD) epithelium. In the present study, we aimed to investigate the subtypes of immune cell infiltrates in HMD and low MD (LMD) breast tissue.

**Methods:**

Fifty-four women undergoing prophylactic mastectomy at Peter MacCallum Cancer Centre or St. Vincent’s Hospital were enrolled. Upon completion of mastectomy, HMD and LMD areas were resected under radiological guidance in collaboration with BreastScreen Victoria and were subsequently fixed, processed, and sectioned. Fifteen paired HMD and LMD specimens were further selected according to their fibroglandular characteristics (reasonable amount [> 20%] of tissue per block on H&E stains) for subsequent IHC analysis of immune cell infiltration.

**Results:**

Overall, immune cell infiltrates were predominantly present in breast ducts and lobules rather than in the stroma, with CD68^+^ macrophages and CD20^+^ B lymphocytes also surrounding the vasculature. Macrophages, dendritic cells (DCs), B lymphocytes, and programmed cell death protein 1 (PD-1) expression were significantly increased in HMD epithelium compared with LMD. Moreover, significantly higher levels of DCs, CD4^+^ T cells, and PD-1 were also observed in HMD stroma than in LMD stroma. The increased expression of interleukin (IL)-6 and IL-4, with unaltered interferon-γ, indicate a proinflammatory microenvironment.

**Conclusions:**

Our work indicates that the immune system may be activated very early in breast cancer development and may in part underpin the breast cancer risk associated with HMD.

**Electronic supplementary material:**

The online version of this article (10.1186/s13058-018-1010-2) contains supplementary material, which is available to authorized users.

## Background

*Mammographic density* (MD) refers to the percentage of radio-opaque fibroglandular breast tissue on a mammogram [1]. In 1969, Wolfe et al. first proposed that an increased proportion of dense breast tissue might be associated with increased breast cancer (BC) risk [2]; however, evidence was scarce at that time to support this hypothesis. Furthermore, dense breast tissue could present a masking effect for small tumors on mammography, making early cancer detection on mammograms challenging [3]. Hence, it was widely believed that the increased BC risk was in fact secondary to the masking effect, rather than any real difference in cancer development due to MD [4]. However, over the past 20 years, well-powered case-control and cohort studies have consistently shown that increased MD is a strong risk factor for BC, independent of any potential masking effect [5–8]. In particular, McCormack and dos Santos Silva performed a meta-analysis of 14,000 cases and 226,000 noncases from 42 studies and found that the proportion of dense area, or percent MD (PMD), was consistently associated with BC risk [9]. Subsequently, important questions raised were whether BC preferentially arose from tissue of HMD areas, and if so, what are the characteristics of HMD origin BC compared with LMD. Ursin and colleagues showed in a retrospective study that ductal carcinoma in situ (DCIS) lesions were more likely to develop from HMD than from LMD areas of the breast in 28 women, by comparing mammograms at BC diagnosis with the women’s previous mammograms [10]. Other studies also found that BCs arising in HMD regions are more likely to demonstrate features that suggest poor prognosis than those that arise in LMD areas [11–13].

The significance of MD-associated BC risk was highlighted by the fact that in 1993, the American College of Radiology developed the Breast Imaging Reporting and Data System (BI-RADS) system, which divides density qualitatively into four categories [14, 15]. More recently, the Density Education National Survivors’ Effort (www.areyoudense.org) in the United States led a high-profile campaign that encouraged women to ask for additional investigations if their breast tissues were reported as mammographically dense [16]. This subsequently led to bold legislation changes in 32 U.S. states to mandate physicians to inform their patients of their MD categories [17, 18].

Although the association of HMD with increased BC risk has now been well established for years, the underlying biological mechanism of this association continues to perplex researchers. Many biological and molecular studies are beginning to unravel the complexities of the biology behind HMD-associated BC risk [19–24]. Using paired HMD and LMD breast tissues from women undergoing prophylactic mastectomy, we and others have found that HMD breast tissue was associated with increased epithelium, stroma, and collagen and decreased fat percentages compared with LMD tissue; furthermore, HMD regions showed increased number of CD45^+^ immune cells in the epithelium [25, 26]. To date, there is little data on the association of MD with immune cell infiltration; however, immune cell infiltration is observed in early-stage BC (proliferative benign disease and DCIS) as well as invasive BC, where numbers can predict prognosis [27]. In this study, we further investigated the innate and adaptive immune cell infiltration and their functional polarization in HMD and LMD normal breast tissue.

## Methods

### Patient accrual

This study was approved by the Peter MacCallum Human Research Ethics Committee (number 08/21) and St. Vincent’s Hospital Animal Ethics Committee (number 049/09). It was conducted in accordance with the Australian National Statement on Ethical Conduct in Human Research. Between 2008 and 2015, 54 women undergoing unilateral or bilateral prophylactic mastectomy at St. Vincent’s Hospital and the Peter MacCallum Cancer Centre consented to tissue collection through the Victorian Cancer Biobank (VCB 10010). The reasons for their mastectomy procedures were confirmed *BRCA1/2* mutation carrier status, other confirmation mutations such as *PTEN* gene mutation, and a strong family or personal past history of BC. Women were excluded from the study if there was suspicion of malignant lesions on radiological investigations or if the breast that the mastectomy was performed on had been diagnosed with BC or DCIS in the past.

### Selection of HMD and LMD regions within the same breast

Upon the completion of mastectomy, the resected breast was immediately sent to the pathology department on ice. Using sterile techniques, 1-cm-thick, craniocaudal breast slices were resected (breadboarding), palpated for suspicious stiffness, and a slice surplus to diagnostic needs was chosen. The breast slice was then X-rayed against a calibration strip using consistent radiological parameters by trained breast radiographers, followed by selection of high and low MD tissue regions for comparative study. The method was detailed in previous studies [19–21, 26, 28, 29]. If the breast slice imaging did not result in clear black and white regions (allowing density differences to be observed) the woman was excluded from this study. The paired HMD and LMD tissues were then fixed in neutral buffered formalin, before undergoing tissue processing, embedding and sectioning at 4 μm thickness for subsequent staining.

### IHC staining

Conventional H&E staining was performed on all paired high and low MD tissues. On the basis of H&E-stained slides, 15 paired high and low MD tissues were further selected for a focused examination of immune cell influx because they showed abundance of epithelial-stromal areas that represent the characteristics of mammary specimens.

These 15 paired high and low MD tissues underwent successive IHC staining for immune cell analyses. Manual staining was performed for pan-macrophage marker CD68 (1:200, Dako clone PG-M1, code M0876; Agilent Technologies, Santa Clara, CA, USA) and B lymphocyte marker CD20cy (1:100, Dako clone L26, code M0755; Agilent Technologies). A diaminobenzidine (DAB) IHC autostainer (Ventana Medical Systems, Tucson, AZ, USA) was used for dendritic cell (DC) marker CD11c (1:25, clone 5D11; Cell Marque, Rocklin, CA, USA); programmed cell death protein 1 (PD-1) (1:100, clone NAT105; Abcam, Cambridge, MA, USA); helper CD4^+^ T cell (1:50, AP20210 PU-N; Novus Biologicals, Littleton, CO, USA); cytotoxic CD8^+^ T cells (1:50, orb 10325; Biorbyt, San Francisco, CA, USA); cytokines IL-4 (1:500, AB9622; Abcam) IL-6 (1:800, AB6672; Abcam), and interferon (IFN)-γ (1:500, AB25101; Abcam), and natural killer cell (NK) marker CD56 (MRQ-42, Ventana® catalogue no. 760-2625; Cell Marque). For each staining, human benign tonsil tissue was used as a positive control, and no primary antibody was used as a technical negative control. In the case of IL-6, only nine paired samples were used because the other six pairs did not have enough tissue left following staining for the other immune cell markers.

Five-micrometer paraffin-mounted tissue sections were dewaxed and underwent antigen retrieval using either citrate buffer (CD68, CD11c, CD56, CD20cy, IL-4, and IL-6) or ethylenediaminetetraacetic acid (EDTA) (CD4, CD8, PD-1, and IFN-γ) and then incubated with the appropriate primary antibodies followed by biotinylated secondary antibodies. The VECTASTAIN Elite ABC kit (Vector Laboratories, Burlingame, CA, USA) with Dako DAB peroxidase (Agilent Technologies) used as chromogen.

For CD3 and PD1 double staining, we performed Opal multiplex imaging (PerkinElmer, Waltham, MA, USA) according to the manufacturer’s instructions. In brief, 3-μm formalin-fixed, paraffin-embedded sections were deparaffinized and then stained with rabbit monoclonal anti-CD3 (clone SP7; Spring Bioscience, Pleasanton, CA, USA) and a rabbit monoclonal anti-PD1 (Bio SB, Santa Barbara, CA, USA). Antigen retrieval was performed in high-pH EDTA in a pressure cooker for the first antibody and then EDTA in the microwave for subsequent antibodies. Endogenous peroxidases were blocked using hydrogen peroxidase, and following incubation with anti-rabbit secondary antibody, the immunofluorescent signal was visualized using TSA dye 570 or 650 from the Opal™ 7 color fIHC kit (PerkinElmer). All sections were counterstained with Spectral DAPI. Slides were imaged on the Vectra® 3.0 Automated Quantitative Pathology Imaging System (PerkinElmer). Color separation, tissue and cell segmentation, and phenotyping were performed using inForm® software version 2.2 (Perkin Elmer) to extract image data.

### Histological review and digital image analysis

All slides were examined using digital microscopy (AxioVision photomicroscope; Carl Zeiss Microscopy, Thornwood, NY, USA) for punctate brown cytoplasmic staining of each individual immune cell marker. Four random glandular-stromal areas within each tissue sample were photographed at × 40 magnification. The number of positively stained immune cells and the total number of cells within each histological compartment (epithelial and stromal regions) were manually counted for each image. Thus, for the immune cell counts in the epithelial area, the total number of epithelial cell nuclei was counted on the selected region, and then the number of positively stained cells was counted. The number of positive cells was then presented as a percentage of total epithelial cells. Scoring was performed in a blinded manner. The percentage of positive staining was calculated as an average value derived from results of all four images for each tissue sample. For stroma, only cells with elongated nuclei and positive cytoplasmic brown staining were counted as positive stains.

### Statistical analysis

For each immune cell marker staining, the data were first assessed for equal variance using a normality test. If data passed the normality test, a paired *t* test was used to analyze the percentage of positive immune cell staining in the epithelium and the stroma, respectively. When data were not normally distributed, a nonparametric Wilcoxon matched-pairs rank test was used. Outliers were identified using Grubbs’ test. A conventional two-tailed alpha level of 0.05 was used to define statistical significance.

## Results

### Characteristics of the study population and tissue samples

The mean age of the 15 selected women was 46 years. The majority had borne children and were premenopausal (9 of 15). Over half (8 of 15) had confirmed *BRCA1/2* mutation carrier status, with or without strong past or family history of BC. Their BI-RADS scores were evenly spread across the four categories. Their characteristics are summarized in Table 1 and are representative of the entire cohort of 54 patients accrued. Of the 54 women, 14 had a BI-RADS score of 3, and 10 had a score of 4, giving an overall 44.0% of patients with heterogeneously or extremely dense breasts. For the 15 women included in our study, 3 of 15 had a BI-RADS score of 4, and 4 of 15 had a BIRADS score of 3. Thus, 46% of patients had heterogeneously or extremely dense breasts, representative of the entire cohort.

**Table 1:** Demographic characteristics of study participants

Selected characteristics	Number or mean
Age at surgery date	Mean 46 years (range, 31–64 years)
BI-RADS category	
4	3
3	4
2	5
1	3
Risk factors *(some women had > 1 risk factor)*	
Strong family history	11
BRCA1^+^	3
BRCA 2^+^	5
Past history of BC or DCIS	10
Menopausal status	
Premenopausal	9
Perimenopausal	1
Postmenopausal	5
Parity	
Parous	11
Nulliparous	4

*Abbreviations: BI-RADS* Breast Imaging Reporting and Data System, *BC* Breast cancer, *DCIS* Ductal carcinoma in situ

BI-RADS score 1 = predominantly fat, 2 = scattered fibroglandular densities, 3 = heterogeneously dense, 4 = extremely dense

### HMD breast tissue has an increased infiltration of innate and adaptive immune cells

**Fig. 1:**
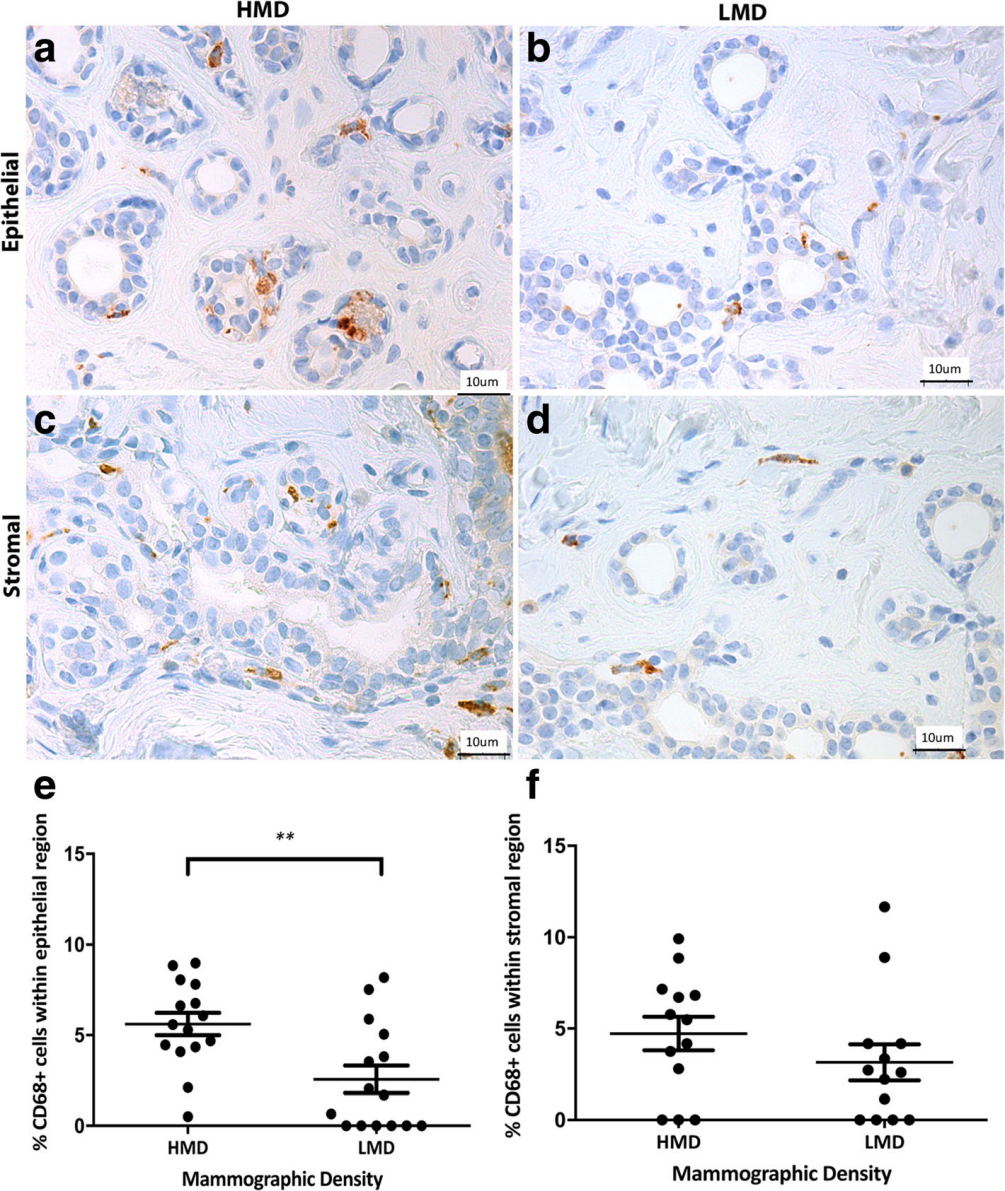
Analyses of CD68 IHC staining. Representative photomicrographs of epithelial (**a, b**) and stromal regions (**c, d**) from tissue specimens resected from HMD (**a, c**) and LMD (**b, d**) regions, respectively. **e** and **f** Quantification of all samples for cells in the epithelium (**e**) and stroma (**f**). **p < 0.01. Scale bar = 10 μm. All error bars indicate SEM. *HMD* High mammographic density, *LMD* Low mammographic density

**Fig. 2:**
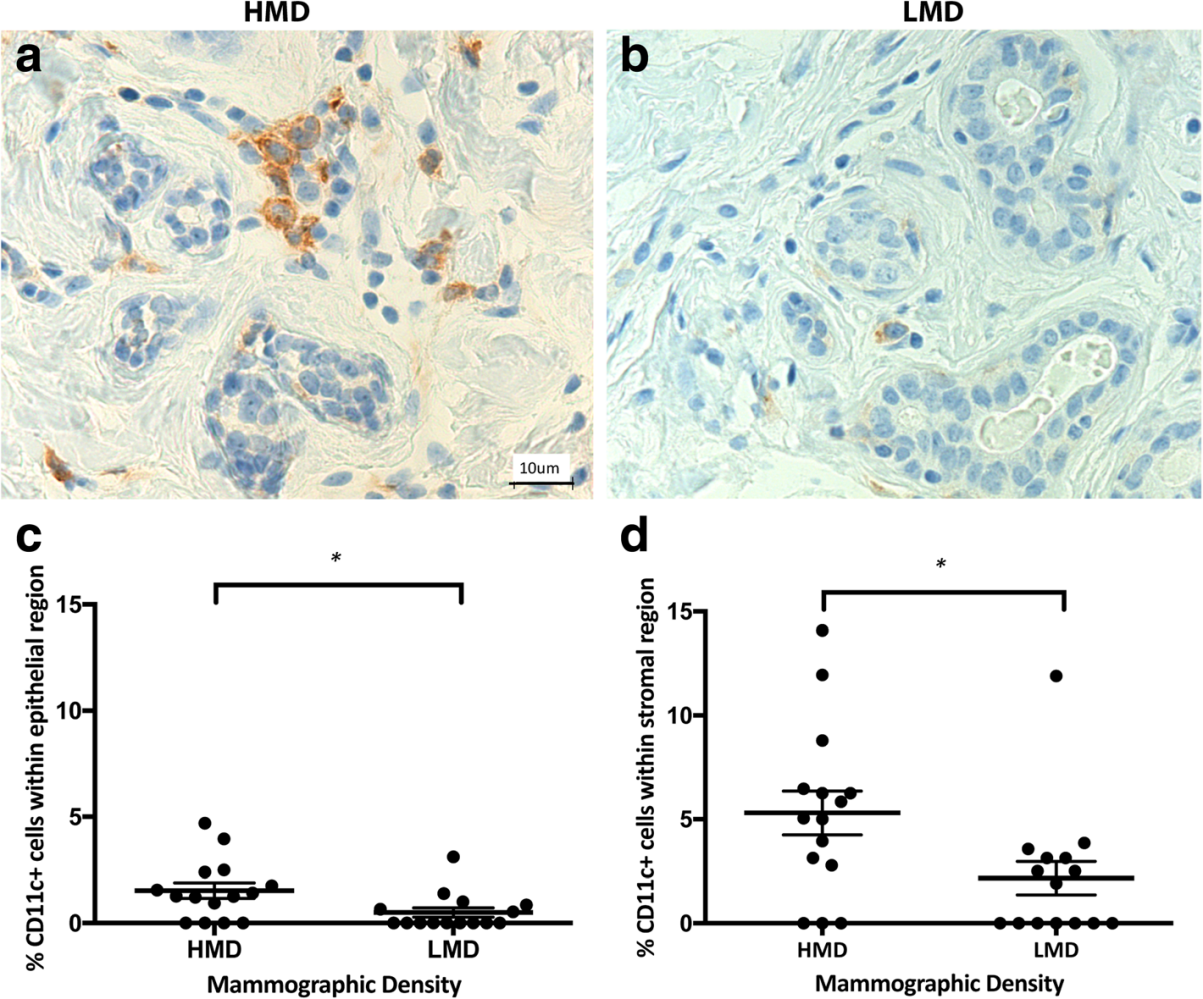
Analyses of CD11c IHC staining. Representative photomicrographs of epithelial and stromal regions (**a, b** show both) from tissue specimens resected from HMD (**a**) and LMD (**b**) regions, respectively. **c** and **d** Quantification of all samples for cells in the epithelium (**c**) and stroma (**d**). *p < 0.05. Scale bar = 10 μm. All error bars indicate SEM. *HMD* High mammographic density, *LMD* Low mammographic density

**Fig. 3:**
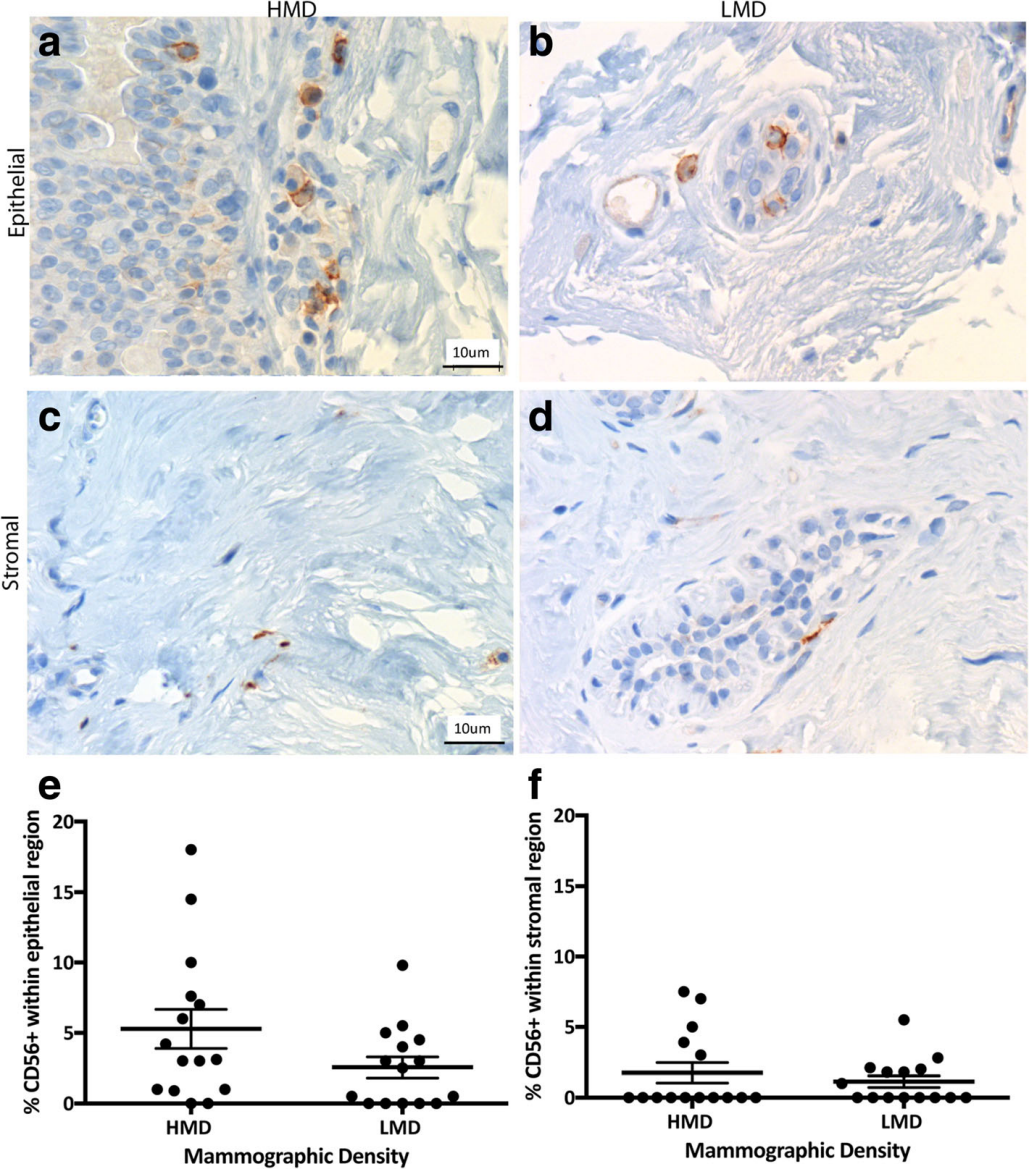
Analyses of CD56 IHC staining. Representative photomicrographs of epithelial (**a, b**) and stromal regions (**c, d**) from tissue specimens resected from HMD (**a, c**) and LMD (**b, d**) regions, respectively. **e** and **f** Quantification of all samples for cells in the epithelium (**e**) and stroma (**f**). Scale bar = 10 μm. All error bars indicate SEM. *HMD* High mammographic density, *LMD* Low mammographic density

Having shown previously that CD45^+^ immune cells were more frequent in HMD than in LMD samples [26], in the present study we assessed which innate and adaptive immune cells were increased. The innate immune cells assessed included macrophages, DC, and NK cells. CD68^+^ macrophages were observed in both epithelial and stromal areas in high and low MD tissue with approximately equal numbers in each cellular compartment. The parenchymal macrophages were present in both lobules and ducts, and they were scattered in both basal and luminal layers of the epithelium. In the epithelium, there were significantly more CD68^+^ cells in HMD regions than in LMD regions (*p* = 0.004) (Fig. 1). CD11c^+^ DC were present in the breast epithelium (lobules and ducts) as well as stroma (Fig. 2). The percentage of DC was significantly higher in HMD than in LMD regions (*p* < 0.05) in the epithelium and stroma (Fig. 2). CD56^+^ NK cells were present in the breast epithelium and stroma, with approximately twofold higher levels in the former. There was no significant difference in NK cell numbers between HMD and LMD tissue in either epithelial or stromal areas (Fig. 3).

**Fig. 4:**
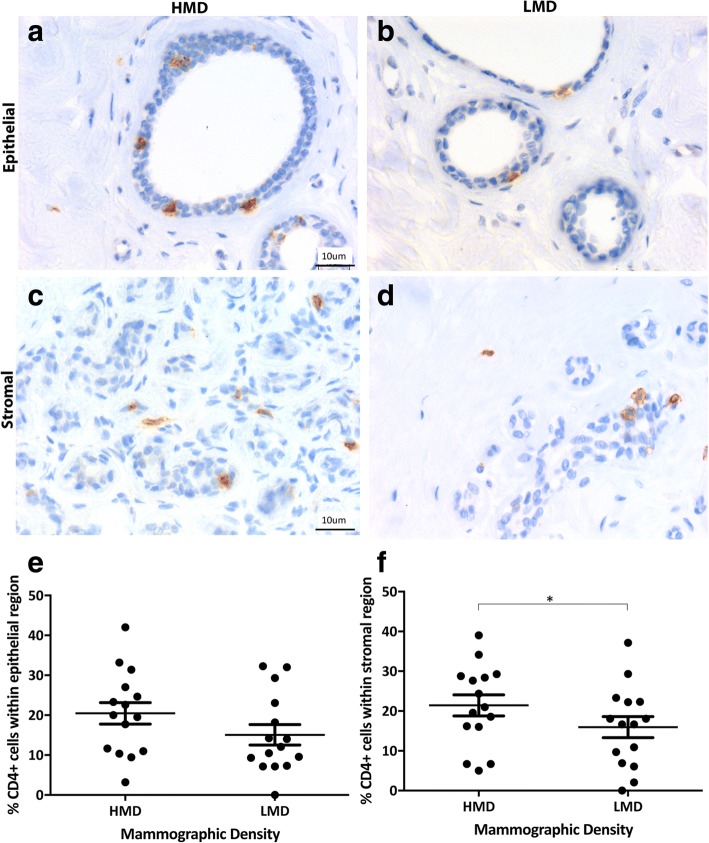
Analyses of CD4 IHC staining. Representative photomicrographs of epithelial (**a, b**) and stromal regions (**c, d**) from tissue specimens resected from HMD (**a, c**) and LMD (**b, d**) regions, respectively. **e** and **f** Quantification of all samples for cells in the epithelium (**e**) and stroma (**f**). *p < 0.05. Scale bar = 10 μm. All error bars indicate SEM. *HMD* High mammographic density, *LMD* Low mammographic density

**Fig. 5:**
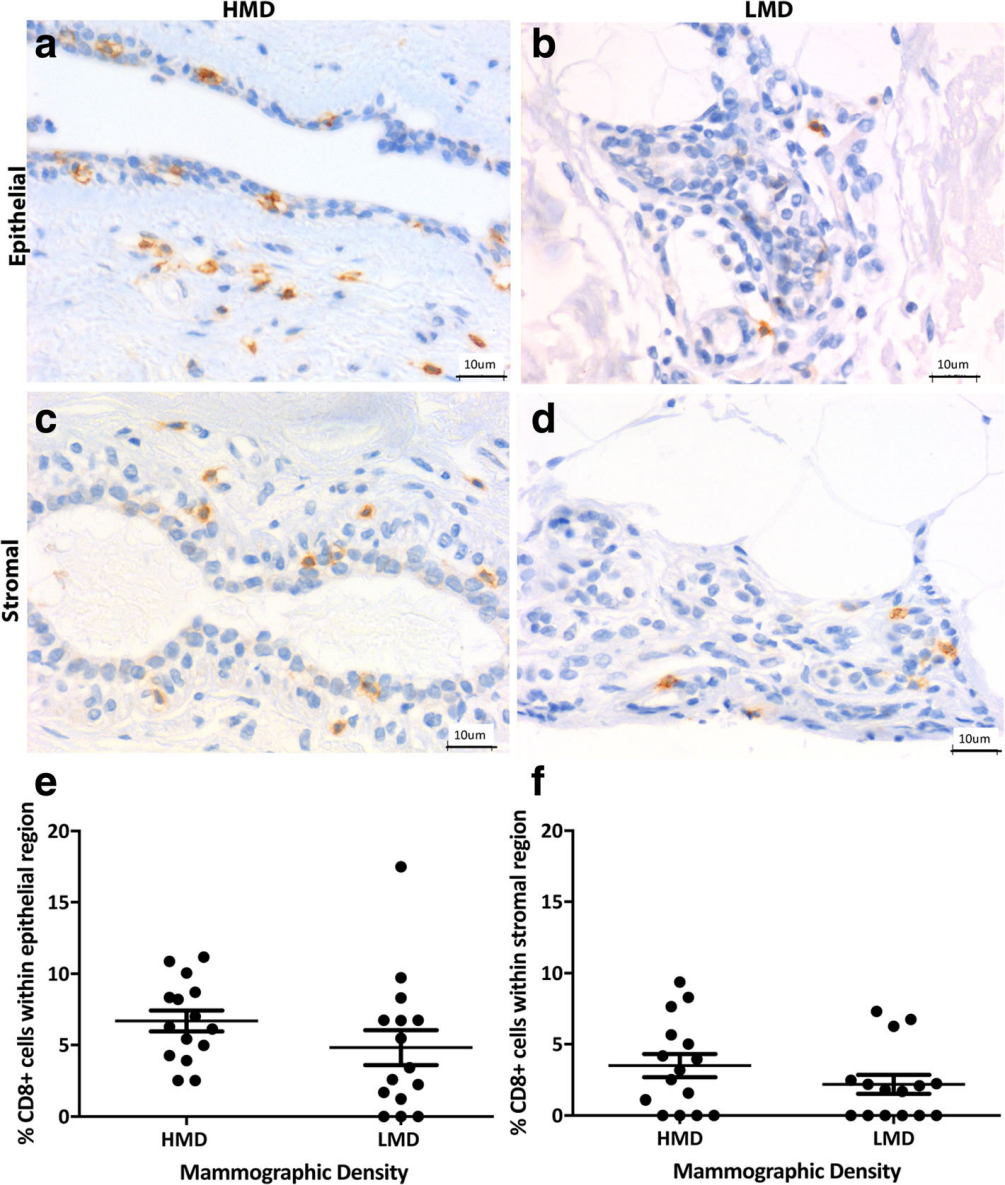
Analyses of CD8 IHC staining. Representative photomicrographs of epithelial (**a, b**) and stromal regions (**c, d**) from tissue specimens resected from HMD (**a, c**) and LMD (**b, d**) regions, respectively. **e** and **f** Quantification of all samples for cells in the epithelium (**e**) and stroma (**f**). Scale bar = 10 μm. All error bars indicate SEM. *HMD* High mammographic density, *LMD* Low mammographic density

**Fig. 6:**
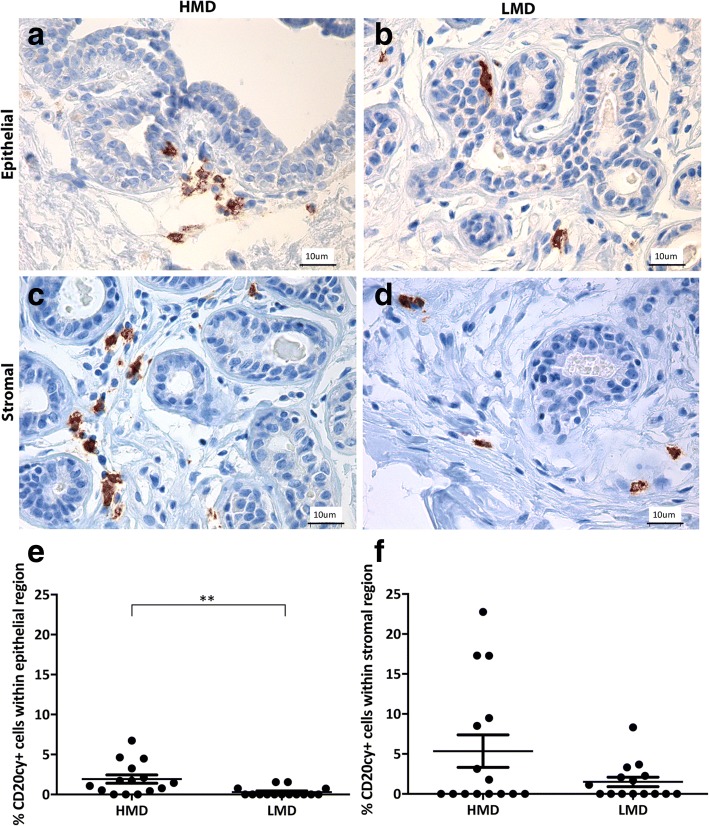
Analyses of CD20cy IHC staining. Representative photomicrographs of epithelial (**a, b**) and stromal regions (**c, d**) from tissue specimens resected from HMD (**a, c**) and LMD (**b, d**) regions, respectively. **e** and **f** Quantification of all samples for cells in the epithelium (**e**) and stroma (**f**). **p < 0.01. Scale bar = 10 μm. All error bars indicate SEM. *HMD* High mammographic density, *LMD* Low mammographic density

Adaptive immune cells assessed included B cells and T-cell subsets (CD4^+^ and CD8^+^). We observed a trend for increased CD4^+^ T cells in the epithelium (*p* = 0.057) of HMD tissues and significantly more CD4^+^ T cells in the HMD stroma than in LMD areas (*p* < 0.05) (Fig. 4). The percentage of CD8^+^ cytotoxic T cells was similar in the epithelium and stroma of HMD and LMD tissues (Fig. 5). The mean CD4/CD8 ratio in the stroma of HMD was 10:1, and in LMD it was 9.7:1, which was not significantly different (*p* = 0.76). Within the epithelium, the CD4/CD8 ratio was 3:1 in the HMD and 5:1 in LMD and again was not significantly different (*p* = 0.52). CD20cy^+^ B cells were present in the epithelium and stroma (Fig. 6). Within the epithelium, the number of B cells in HMD tissue was significantly higher than in LMD tissue (*p* = 0.004). In the stroma, there was a modest but nonsignificant trend for increased CD20cy^+^ B-cell infiltration in HMD compared with LMD tissue.

### HMD environment shows immune activation and protumor cytokines

**Fig. 7:**
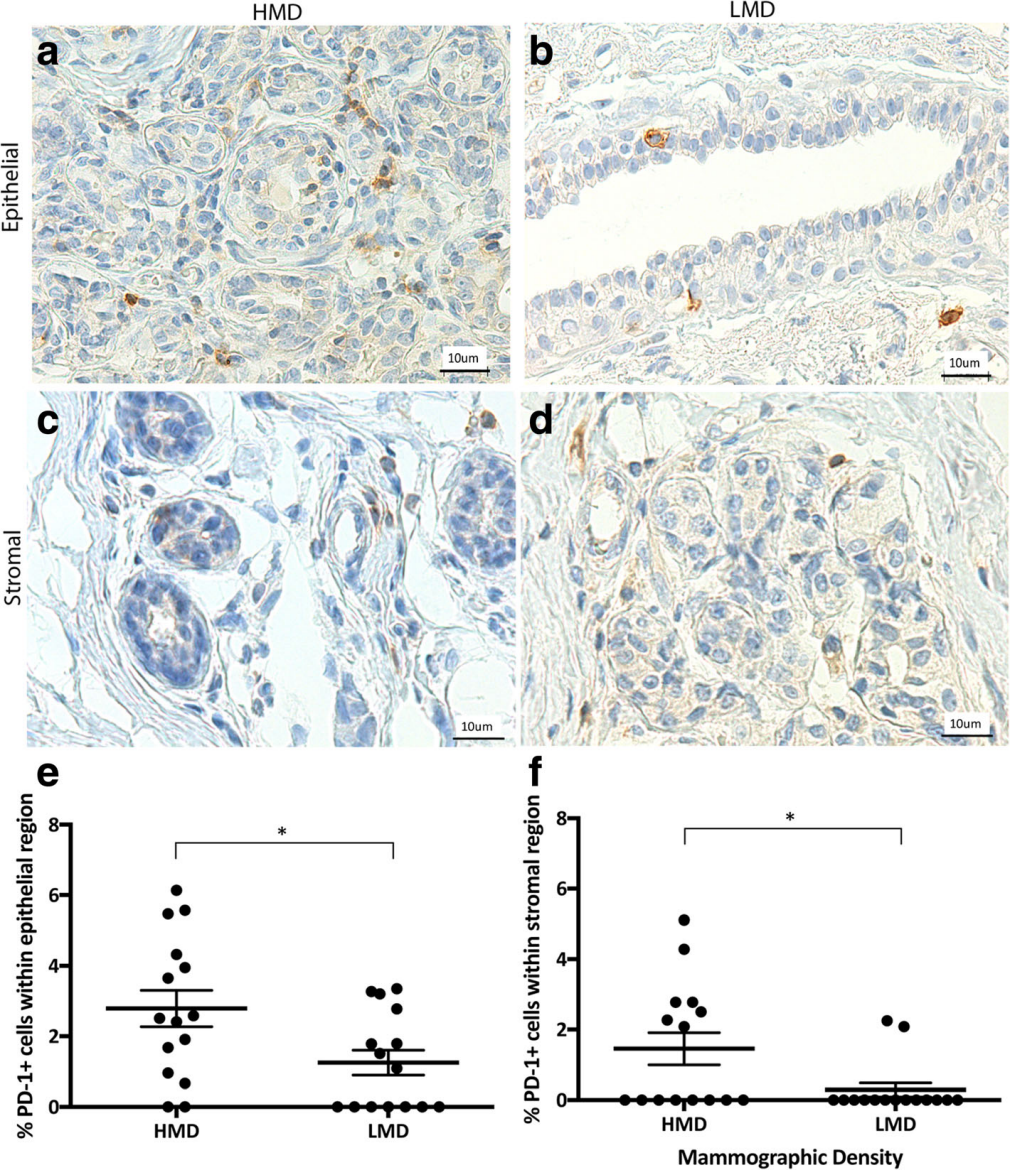
Analyses of programmed cell death protein 1 (PD-1) IHC staining. Representative photomicrographs of epithelial (**a, b**) and stromal regions **(c, d**) from tissue specimens resected from HMD (**a, c**) and LMD (**b, d**) regions, respectively. **e** and **f** Quantification of all samples for cells in the epithelium (**e**) and stroma (**f**). *p < 0.05. Scale bar = 10 μm. All error bars indicate SEM. *HMD* High mammographic density, *LMD* Low mammographic density

To explore whether the T cells present were activated, we examined PD-1 expression. The number of PD-1^+^ cells (1–2%) that we observed was low; however, the numbers of PD-1^+^ cells in the epithelium and stroma were significantly higher in HMD than in LMD tissue (*p* < 0.05) (Fig. 7). Further staining using Opal multiplex imaging revealed that the PD-1-positive cells were CD3^+^ T cells (Additional file 1: Figure S1).

**Fig. 8:**
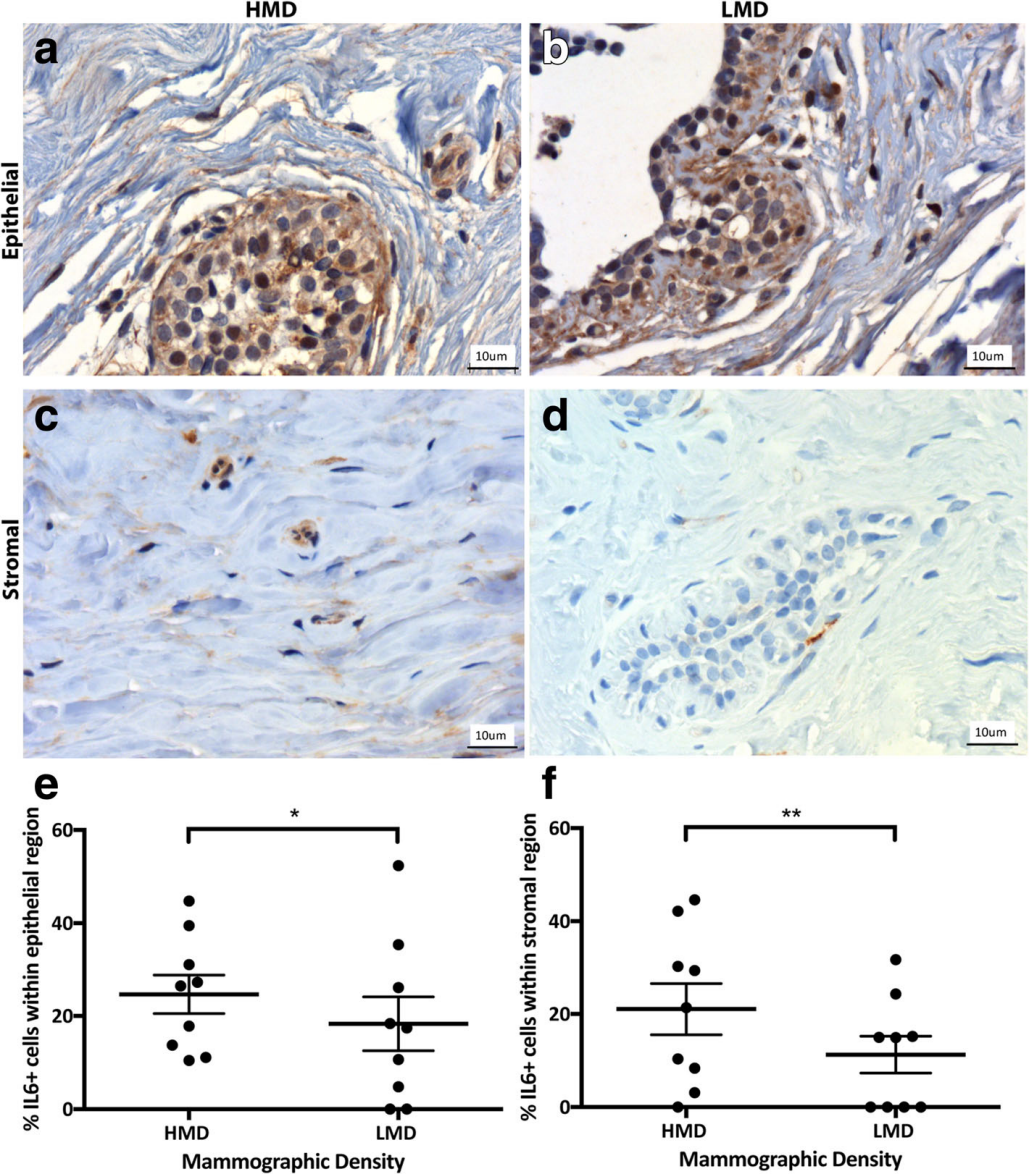
Analyses of interleukin (IL)-6 IHC staining. Representative photomicrographs of epithelial (**a, b**) and stromal regions (**c, d**) from tissue specimens resected from HMD (**a, c**) and LMD (**b, d**) regions, respectively. **e** and **f** Quantification of all samples for cells in the epithelium (**e**) and stroma (**f**). *p < 0.05, **p < 0.01. Scale bar = 10 μm. All error bars indicate SEM. *HMD* High mammographic density, *LMD* Low mammographic density

**Fig. 9:**
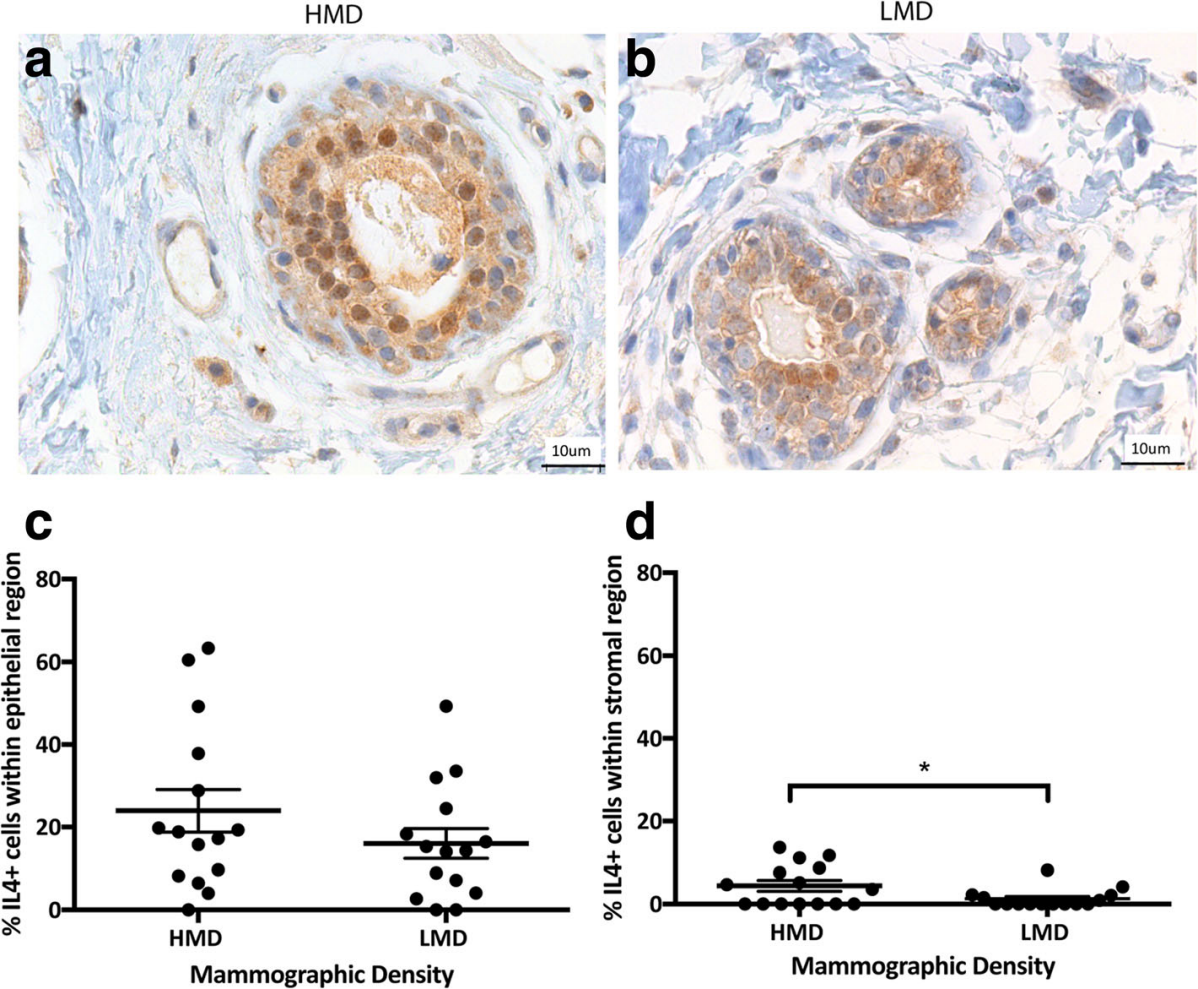
Analyses of interleukin (IL)-4 IHC staining. Representative photomicrographs of epithelial and stromal regions (images show both regions) from tissue specimens resected from HMD (**a**) and LMD (**b**) regions, respectively. **c** and **d** Quantification of all samples for cells in the epithelium (**c**) and stroma (**d**). *p < 0.05. Scale bar = 10 μm. All error bars indicate SEM. *HMD* High mammographic density, *LMD* Low mammographic density

**Fig. 10:**
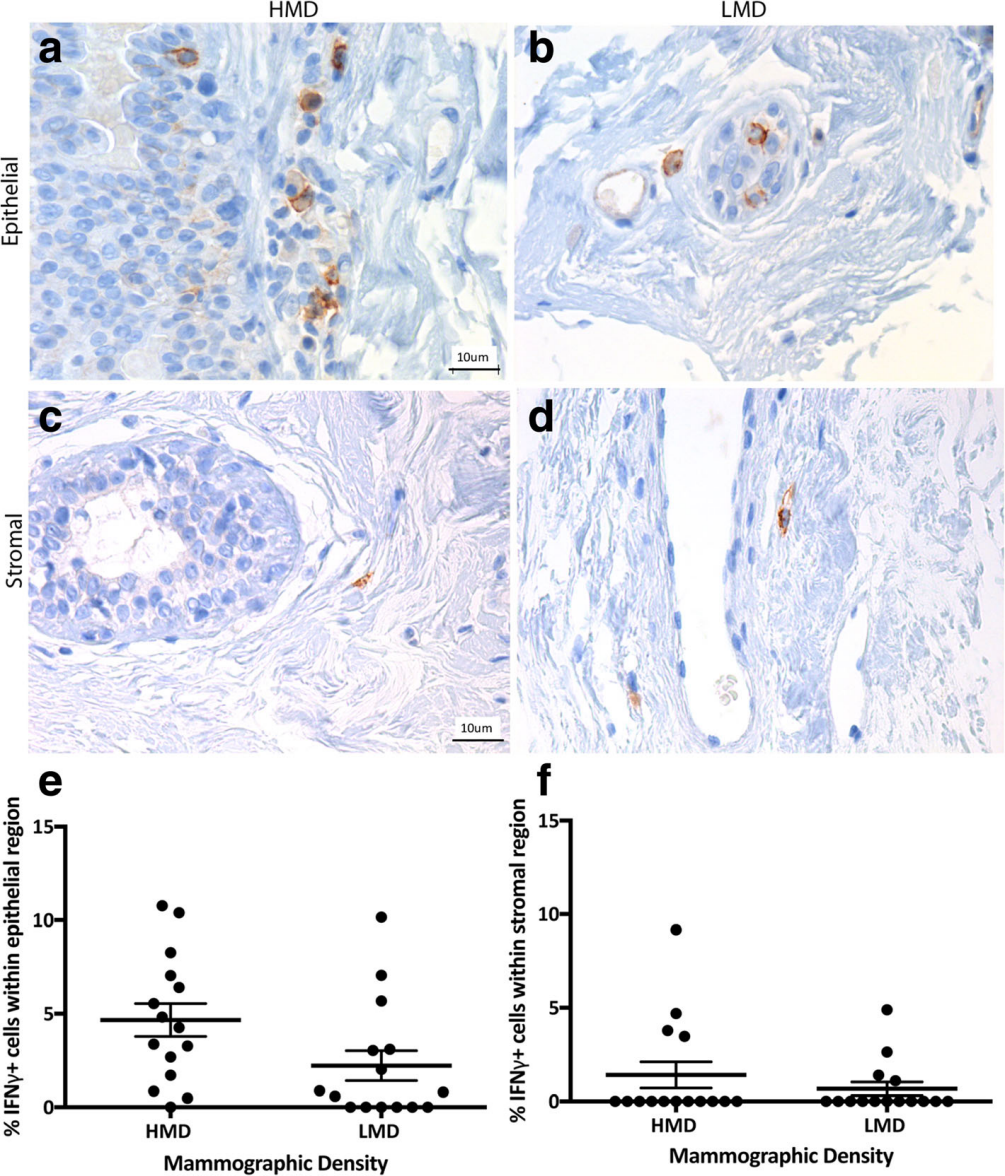
Analyses of interferon (IFN)-γ IHC staining. Representative photomicrographs of epithelial (**a, b**) and stromal regions (**c, d**) from tissue specimens resected from HMD (**a, c**) and LMD (**b, d**) regions, respectively. **e** and **f** Quantification of all samples for cells in the epithelium (**e**) and stroma (**f**). Scale bar = 10 μm. All error bars indicate standard error of the mean (SEM. *HMD* High mammographic density, *LMD* Low mammographic density

We next examined immune cell functional polarization by examining signature cytokines for inflammation (IL-6) and either a Th1 (IFN-γ) or Th2 (IL-4) response. In the breast, the percentage of IL-6^+^ cells ranged from a mean of 18–24% in the epithelium and 11–21% in the stroma (Fig. 8). In the epithelium and stroma, IL-6^+^ cells were significantly increased in HMD areas compared with LMD areas (*p* < 0.05 and *p* < 0.01, respectively). IL-4^+^ cells ranged from a mean of 16–24% in the epithelium and 1–4% in the stroma (Fig. 9); however, there was no significant difference in IL-4^+^ cell numbers between HMD and LMD. In the stroma, the percentage of IL-4^+^ cells was significantly higher in HMD than in LMD tissue (*p* < 0.05) (Fig. 9). Of note, some epithelial cells appeared to be positive for IL-4 and IL-6, which concurs with the Human Protein Atlas databank (www.proteinatlas.org). The percentage of IFN-γ^+^ cells in the breast was 2–4% in the epithelium and 0.67–1.41% in the stroma (Fig. 10). In the breast epithelium, there was a nonsignificant trend (*p* = 0.06) for an increase in IFN-γ^+^ cells in HMD compared with LMD; however, there was no difference in the stroma (Fig. 10).

IFN-γ signaling is known to enable activation of the PD-1 signaling axis [30], so we assessed whether the expression of IFN-γ in our MD samples correlated with PD-1 expression in the same samples. There was a moderate positive correlation between IFN-γ and PD-1 (*r* = 0.476, *p* = 0.004) (Additional file 2: Figure S2).

Recently, benign breast disease tissue has been shown to have higher densities of multiple immune cell types, especially macrophages and DC, compared with normal breast tissues [31]. To determine if the increased immune cell infiltration in HMD tissue was due to the presence of benign breast lesions, we scored all sections for evidence of nonproliferative disease, proliferative disease without atypia, or the presence of atypical hyperplasia (atypical ductal hyperplasia or atypical lobular hyperplasia). We found no evidence of any of these lesions. In addition to this, we assessed columnar cell hyperplasia, flat epithelial atypia, sclerosing adenosis, cysts, unusual ductal hyperplasia, calcification or fibroadenoma, and duct ectasia. We observed only one HMD sample with evidence of a small amount of sclerosing adenoma, and its matching LMD had a very small focal region of columnar cell change. We identified one other HMD sample with a small amount of sclerosing adenosis. Another HMD sample with a very focal amount of columnar cell change was also observed (Additional file 3: Table S1). Because there was only a very small amount of benign change present in a minority of samples, and because those samples did not harbor the strongest increase in immune cells, we concluded that benign features do not account for the increase in immune cells in HMD tissue.

Because our tissues were obtained from prophylactic mastectomies and a significant proportion of our patients had germline mutations in known predisposition genes (*BRCA1/2*), we assessed whether the immune changes differed in those with *BRCA1/2* mutations compared with those without. Although our study was not appropriately powered for such an analysis, we found that the trend was similar in both nonmutation carriers and *BRCA1* mutation carriers. In many cases, the changes were more evident in the nonmutation carriers. *BRCA2* carriers shared similar changes according to density with the nonmutation carriers and *BRCA1*, but not for all immune cells (Additional file 4: Figure S3).

## Discussion

Following our work identifying an increase in the immune cells (CD45^+^vimentin^+^cytokeratin^−^ cells) within the epithelial region of dense breast tissue [26], in the present study we show dense breast tissue has increased parenchymal macrophages, DC, and B cells, but not NK cells. HMD tissue also has a trend for increased CD4^+^ T cells and a significant increase in expression of the checkpoint inhibitor PD-1, confirming that the T cells present are activated. IL-6 was increased in the epithelial regions, and both IL-6 and IL4 were increased in the stromal regions of HMD samples, but not IFN-γ. This suggests that the preneoplastic breast tissue of women with high breast density has increased numbers of innate and adaptive immune cells, with a proinflammatory functional polarization consistent with the increased risk of developing BC that has been associated with HMD.

BC has largely not been considered immunogenic, because incidence is not increased in patients who are immune-suppressed; however, there are now irrefutable data demonstrating that the immune cell infiltrate of a breast tumor affects its growth and metastasis [27]. In addition, progression from normal to preinvasive and invasive BC has been associated with increases in T cells, B cells, and macrophages [32, 33]. Degnim and colleagues assessed 11 normal breast samples and found increased T- and B-cell numbers in breast lobules with lobulitis and found that immune cells were present mainly in breast lobules rather than in the stroma and that cytotoxic T cells and DC were integrated within the epithelium [34]. We also found macrophages, DC, NK cells, B cells, and T cells in normal breast tissue. Furthermore, we showed the expression of PD-1 and secretion of IL-6, IL-4, and IFN-γ in glands and stroma of normal breast, suggesting that there is a dynamic breast immune surveillance system. Similarly, Degnim and colleagues assessed benign breast disease tissue and found that this tissue had higher densities of multiple immune cell types, especially macrophages and DC, compared with normal breast tissues [31]. Although our HMD tissue did not show signs of benign breast disease, we also found increased levels of these two innate immune cells, indicating that they may be important for the early immune response to breast changes that may, in some cases, develop into lesions.

We found that the number of innate immune cells was increased in HMD samples compared with LMD samples. Macrophages are phagocytic cells that act to maintain immune surveillance within tissues and constantly survey their surroundings for signs of tissue damage or invading organisms. They stimulate lymphocytes and other immune cells to respond when danger signals are phagocytosed and/or detected by cell surface receptors [35]. In the present study, we show that macrophages were increased in the epithelial regions of HMD samples. Previously, we reported that epithelial macrophage numbers were not altered with density [26]; however, only nine women and three random areas of each section were assessed. The larger sample sizes and increased areas used in the present study allowed significant macrophage changes to be revealed. The increased epithelial macrophages support our recent finding that chemokine ligand 2 (CCL2) or monocyte chemoattractant protein 1 (MCP-1) is significantly increased in HMD epithelium [36]. CCL2 recruits macrophages but also stimulates protumorigenic M2 macrophage polarization [36, 37], which is supported by the increased epithelial IL-6 expression that we report here, as well as its role in M2 orientation [38].

DC are powerful antigen-presenting cells [39] with molecular sensors enabling them to sense danger and to transmit this to lymphocytes to initiate the T-cell immune response [40] and aid in tumor cell death. We found increased DC in HMD epithelial and stromal compartments compared with LMD, suggesting increased antigen presentation and hence potentially enhanced immune surveillance. Because the generation of tumor-specific T cells relies on the ability of mature DCs to cross-present tumor antigens, future studies will need to assess the functional status of DC to fully understand the implications of increased DC. NK cells are tumor cell- and virus-killing innate immune cells that do not need to match with a major histocompatibility complex (MHC) subclass, the way CD8^+^ T cells do [41]. NK cell dysfunction has been associated with BC progression [42]. We found no significant difference in the percentage of NK cells between high and low MD, suggesting that NK cells do not play a key role in the inflammatory microenvironment of high breast density.

In addition to the changes in the innate immune cells, we found changes in the adaptive immune cells within high-density breast tissue. The percentage of B lymphocytes was significantly increased in HMD epithelium. B cells secrete antibodies and inflammatory cytokines and can recognize antigens, regulate antigen processing and presentation, and mount and modulate T-cell and innate immune responses. B-cell infiltration was been associated with worse outcome in patients with metastatic ovarian cancer and progression of orthotopic tumors in mice [43], and their numbers increase with the progression of normal breast and benign proliferative disease through to DCIS and invasive ductal carcinoma [33]. We postulate that the increased number of B cells in HMD may reflect changes in the breast immune surveillance and possibly increased differentiation of B regulatory cells, which themselves can drive T-regulatory differentiation of CD4^+^ T cells.

The CD4^+^ T lymphocytes were markedly increased in the HMD stroma, whereas no significant difference was observed in terms of CD8^+^ T cells in either compartment. CD4^+^ T cells carry out multiple functions, including activation of innate immune cells, B lymphocytes, and cytotoxic T cells, as well as nonimmune cells. They also play a critical role in the suppression of immune reaction. CD4^+^ T cells can be either T-helper cells (Th) or regulatory T cells (Treg), and they are activated through two signals: T-cell receptor on the T cell and an antigenic peptide presented by MHC class II on the antigen-presenting cell requiring a second costimulatory signal [44, 45]. Within the Th cell population, there are a number of subtypes, including Th1, Th2, Th9, Th17, Th22, and ThFH (Follicular T helper), that differ in their functions, signature cytokine profiles, and cell targets [46]. Due to the increased levels of IL-6 and IL-4 in HMD breast tissue (but steady state of IFN-γ), we postulate that the increased CD4^+^ T cells are Th2-oriented because IL-6 drives Th2 differentiation [47, 48] and IL-4 production is enhanced by IL-6. IL-6 has already been associated with breast density in genome-wide association studies on cancer-free breast tissue, where genetic variations in nine tagging single-nucleotide polymorphisms in the IL-6 gene were significantly associated with HMD [49]. Higher transcript expression of IL-6 has also been reported in HMD epithelial areas compared with LMD in BC [50].

The PD-1/PD-L1 pathway is an inhibitory immune checkpoint pathway that is upregulated within the tumor microenvironment [51, 52], and PD-1/PDL-1 checkpoint inhibitors are showing unprecedented clinical efficacy in certain cancer types, especially melanoma and lung [53]. Its normal biological role lies in preventing overstimulation of the immune responses and helping maintain immune tolerance to self-antigens [54, 55]. PD-1 is expressed on activated T cells but also on other immune cells, including activated B cells and NK cells [56] and Tregs [57]. We show that PD-1 expression was increased within the epithelial and stromal regions of HMD samples compared with LMD, suggesting an increased immune self-tolerance in the HMD. The levels of PD-1 in normal human breast (0.29–2.79%) (*see* Table 2) are much lower than in BC, where expression ranges from 19% to 59% [58, 59]. In murine BC studies, tumor-induced CD8^+^ T cells that express a high level of PD-1 were found to be ineffective in controlling tumor growth [60]. Thus, the increased level of PD-1 protein in HMD compared with LMD regions suggests that the function of T cells may be impaired, which may contribute to the increased BC risk that occurs in HMD.

**Table 2:** Overview of all immune cell analyses between high (HMD) and low (LMD) density breast samples

	Epithelium		Difference (*p* value)	Stroma		Difference (*p* value)
Marker	HMD	LMD		HMD	LMD	
CD68	5.61 (0.61)	2.56 (0.75)	***p*** **=0.004**	4.72 (0.9)	3.14 (0.98)	*p*=0.15
CD11c	1.53 (0.36)	0.5 (0.22)	***p*** **=0.02**	5.30 (1.06)	2.16 (0.80)	***p*** **=0.03**
CD56	5.30 (1.41)	2.29 (0.73)	*p*=0.07	1.81 (0.74)	0.92 (0.36)	*p*=0.31
CD20	1.93 (0.52)	0.31 (0.15)	***p*** **=0.0039**	5.35 (2.03)	1.50 (0.59)	*p*=0.28
CD4	20.47 (2.69)	15.07 (2.55)	*p*=0.05	21.42 (2.63)	15.95 (2.64)	***p*** **=0.04**
CD8	6.69 (1.43)	4.82 (1.75)	*p*=0.22	3.49 (0.83)	2.18 (0.66)	*p*=0.15
PD1	2.79 (0.52)	1.25 (0.35)	***p*** **=0.02**	1.45 (0.46)	0.29 (0.20)	***p*** **=0.04**
IL-6	24.68 (4.13)	18.34 (5.80)	***p*** **=0.03**	21.07 (5.53)	11.24 (3.98)	***p*** **=0.006**
IL-4	23.94 (5.19)	16.04 (3.60)	*p*=0.09	4.41 (1.29)	1.28 (0.59)	***p*** **=0.04**
IFNγ	4.66 (0.88)	2.22 (0.80)	*p*=0.06	1.41 (0.69)	0.67 (0.36)	*p*=0.56

Data are presented as the Mean+/- (SEM) percentage of immune + cells (ie: CD68) as a function of total epithelial cell nuclei counted in 4 random areas within each tissue sample. Data with significant differences *p*<0.05 are highlighted bold. N=10-15 women

## Conclusions

Although BC is not classically considered to be immunoresponsive, we are among the first to report a detailed study on the dynamic breast immune environment where HMD tissue is associated with increased infiltration of innate immune cells (macrophages, DC), adaptive immune cells (B cells, CD4 T cells), as well as activated T cells (PD-1 expression) and protumor Th2 polarization (elevated IL-6 and IL4 secretion). This protumorigenic microenvironment may assist an escape from immune regulation for early tumor cell variants.

In the future, assessing the tumor cell-killing function of T cells using in vitro assays and T-cell exhaustion by flow cytometry, as well as exploring DC of different origins using distinct staining markers, may permit further insights into the significance of immune cells associated with HMD. Understanding the interplay of immune cells and their effects on breast epithelium and stroma will provide a novel avenue for BC prevention and treatment that is focused on modulating the immune microenvironment. Future studies will also explore the potential of nonsteroidal anti-inflammatory drugs (NSAIDs) such as aspirin in modulating breast density by normalizing the immune environment. Aspirin is being considered for large-scale prevention studies due to strong data showing a reduced risk of BC occurrence and recurrence [61]. Although there is no evidence to date that NSAIDs can reduce breast density, the existing studies are complicated by the use of varied NSAIDs and the low baseline MD readings of the patients. Tamoxifen has its largest chemopreventative effects in the IBIS-1 (International Breast Cancer Intervention Study I) clinical trials in those women with the largest decrease in breast density, which tended to occur in women with higher baseline levels. Future well-powered clinical studies with women in the highest BI-RADS categories will allow researchers to determine if aspirin can be of benefit to women with high breast density.

## Additional files

Additional file 1: **Figure S1.** PD1 expression occurs in CD3^+^ T cells. Two examples of HMD tissue are shown with PD1 staining in yellow and CD3 staining in red, as well as an overlay of both. DAPI (blue) stains the nuclei. Inset images on the overlays show higher-magnification images of double-positive cells. Scale = 20 μm. (PDF 5214 kb)

Additional file 2: **Figure S2.** Correlation between IFN-γ and PD1 expression in our samples. (PDF 167 kb)

Additional file 3: **Table S1.** Pathological analysis of benign breast lesions within our HMD and LMD cohort. Those patients who did have evidence of benign lesions had only very focal evidence of such. (PDF 1286 kb)

Additional file 4: **Figure S3.** Effect of BRCA mutation status on immune influx. For those immune subsets showing significant differences according to density, we separated the data into those with no mutations (HMD and LMD) and those with confirmed BRCA1 or BRCA2 mutations (e.g., BRCA1 HMD, BRCA1 LMD). Data are expressed as mean ± SEM, **p < 0.01. (PDF 2000 kb)

## Abbreviations

BC: Breast cancer
BI-RADS: Breast Imaging Reporting and Data System
CD4: T helper cells
CD8: Cytotoxic T cells
DCs: Dendritic cells
DCIS: Ductal carcinoma in situ
EDTA: Ethylenediaminetetraacetic acid
HMD: High mammographic density
IFN-γ: Interferon-γ
IL: Interleukin
LMD: Low mammographic density
MD: Mammographic density
MHC: Major histocompatibility complex
NK: Natural killer cells
NSAIDs: Nonsteroidal anti-inflammatory drugs
PD-1: Programmed cell death protein 1
PMD: Percent mammographic density
Treg: T helper regulatory cell
