# Supplementary figures and images for: High mammographic density in women is associated with protumor inflammation
Source: Breast Cancer Res. 2018 Aug 9;20:92. doi: 10.1186/s13058-018-1010-2 (PMC6085707; doi:10.1186/s13058-018-1010-2)

**A**

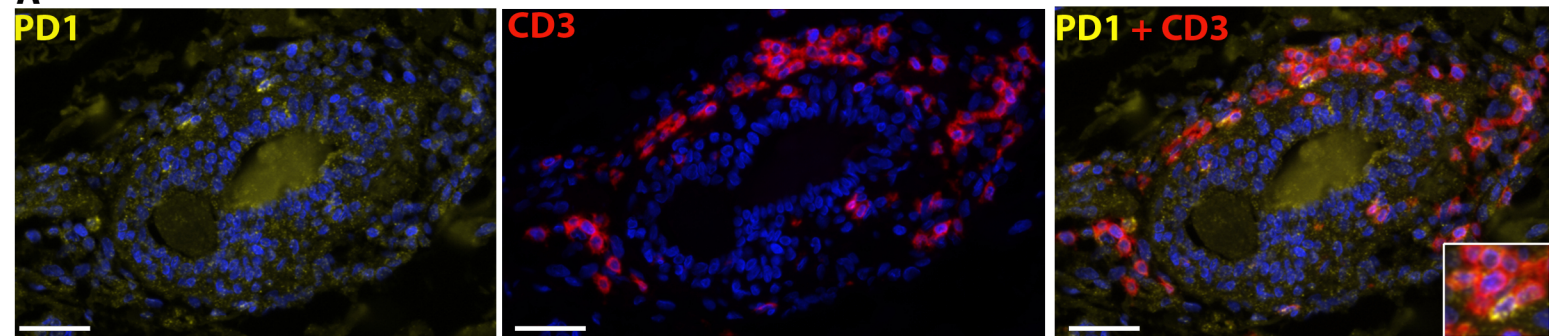

**B**

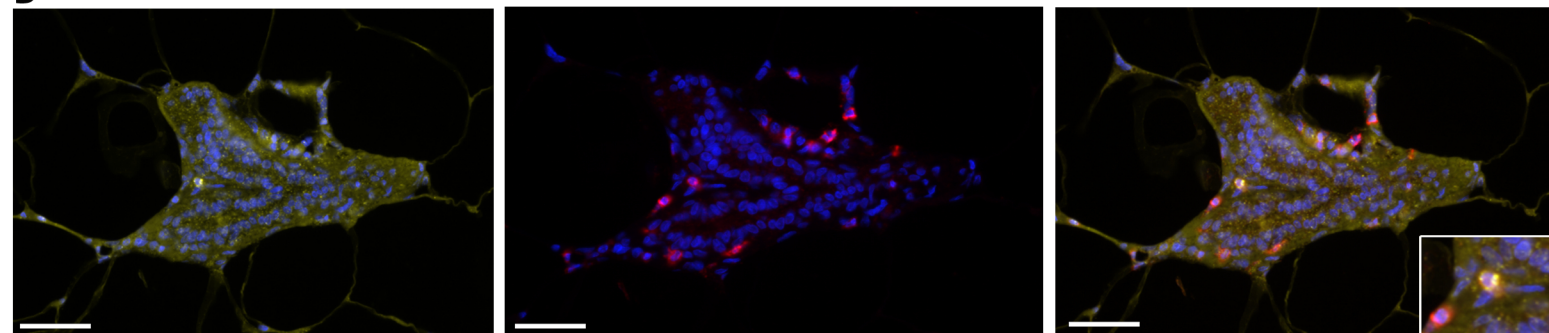

Supplement: Supplementary file 1 — Figure S1. PD1 expression occurs in CD3+ T cells. Two examples of HMD tissue are shown with PD1 staining in yellow and CD3 staining in red, as well as an overlay of both. DAPI (blue) stains the nuclei. Inset images on the overlays show higher-magnification images of double-positive cells. Scale = 20 μm. (PDF 5214 kb) [file 13058_2018_1010_MOESM1_ESM.pdf]

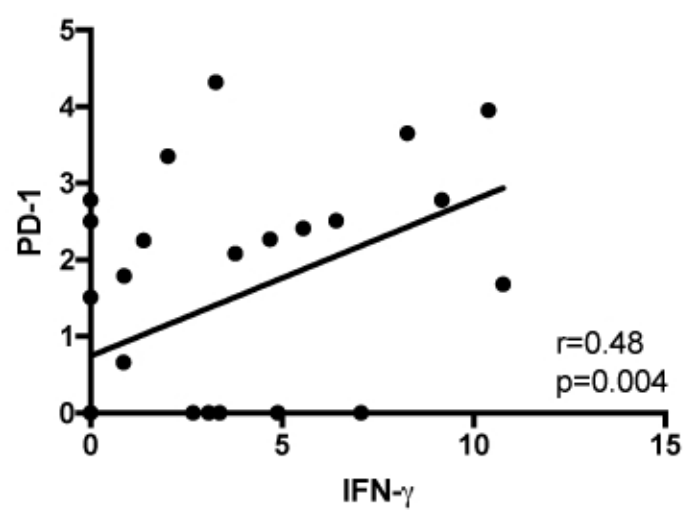

Supplement: Supplementary file 2 — Figure S2. Correlation between IFN-γ and PD1 expression in our samples. (PDF 167 kb) [file 13058_2018_1010_MOESM2_ESM.pdf]

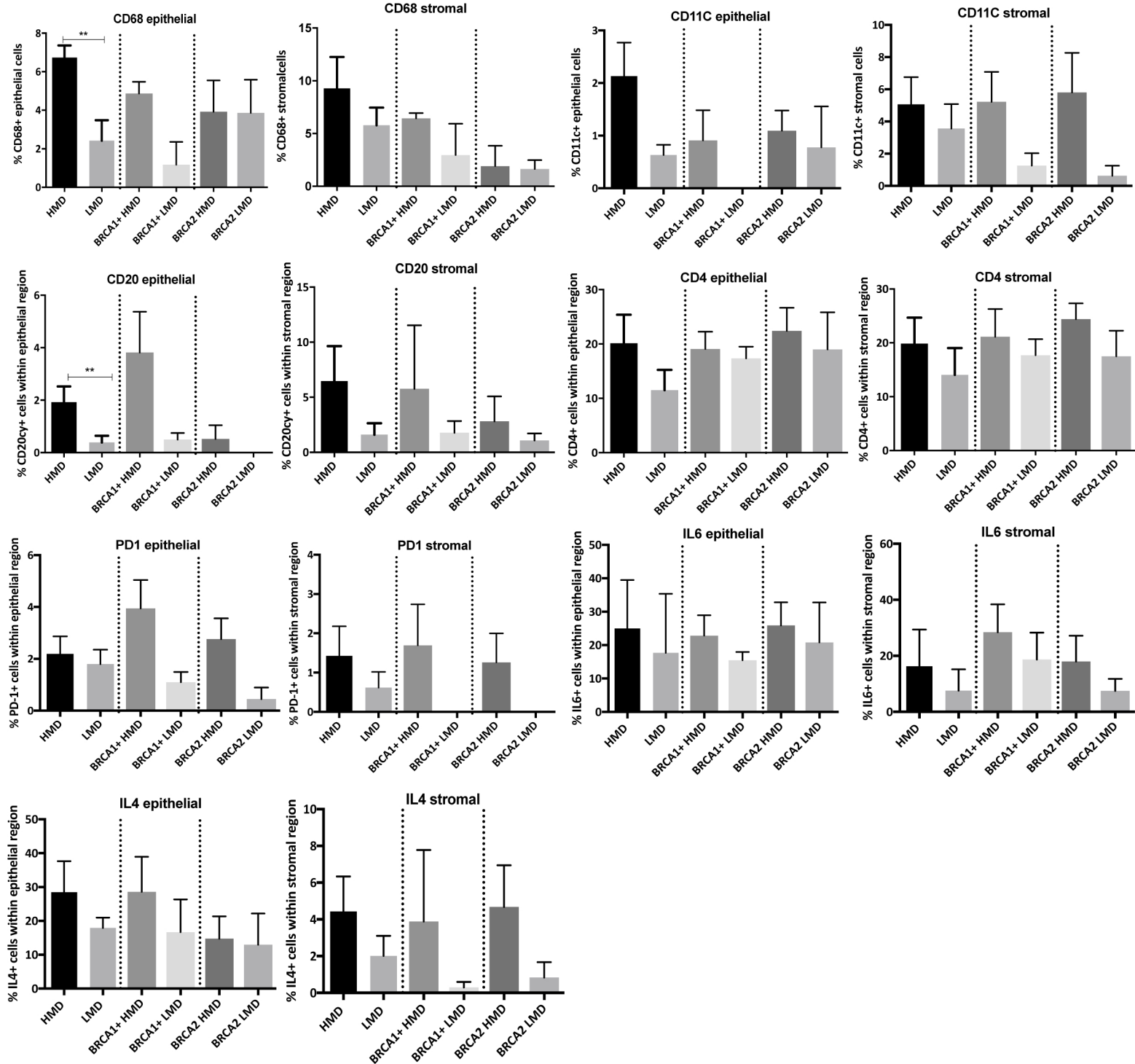

Supplement: Supplementary file 4 — Figure S3. Effect of BRCA mutation status on immune influx. For those immune subsets showing significant differences according to density, we separated the data into those with no mutations (HMD and LMD) and those with confirmed BRCA1 or BRCA2 mutations (e.g., BRCA1 HMD, BRCA1 LMD). Data are expressed as mean ± SEM, **p < 0.01. (PDF 2000 kb) [file 13058_2018_1010_MOESM4_ESM.pdf]
